# Therapeutic Action of Gelsemium

**Published:** 1883-10-20

**Authors:** E. W. Lane

**Affiliations:** Scarboro, Ga.


### THERAPEUTIC ACTION OF GELSEMIUM.

BY E. W. LANE, M.D., SCARBORO, GA.

Will you give me space in your valuable journal that I may say something of the action of the gelsemium *semper virens*, as it has occurred to me in practice. I have been using it for several years, and have been observing its effects as closely as I could in a country practice. I find that it is useful in a great many of the diseases peculiar to this locality. My practice is in what is called a malarious district, and almost all the diseases that our people have, partake of that form. I know not what the malarial poison is, but whether it be cryptogamine, baccilli, poison gasses or something else, I know that the diseases of this locality assume that form in a more or less degree. The nervous system is decidedly out of order, being in a highly irritated condition. I find the gelseminum to be an excellent remedy to quiet the nervous excitement when given in proper doses and at proper intervals. It would be tedious to name all the diseases in which it is useful. It is sufficient to say, that whenever there is an exalted condition of the nervous system, that its use is indicated; the dosing should be just in proportion to the amount of the nervous excitement. If the fever runs high, say up to 103° or 104°, I find it best to give it in such doses as will produce its physiological effect, say twenty-five drops of the saturated tincture in a tablespoonful or two of water, every two hours, until its effect is produced. The physiological effect is to relax the muscular system, and to equalize the circulation by its action upon the cerebro-spinal centres, thus controlling the vasso-motor system of vessels. The skin then becomes moist, and the temperature falls, and the patient is then in a proper condition to take quinine, though not in such heroic doses as some of the profession think best now-a-days, but in four or five grain doses, combined with twelve or fifteen drops of gelsemium every two hours, until the physiological effect of the quinine is produced, and then seldomer, just sufficient to keep up the effect of the quinine.

About twenty years ago, cerebro-spinal meningitis prevailed in this locality to an alarming extent. Some of those who did not die were made blind, and some were partially paralyzed. Since that time it has made its appearance here almost every year, though not to such an alarming extent. We now treat it by first bringing the patient fully under the influence of gelsemium, thus controlling the convulsions, and then using other appropriate remedies, and we seldom lose a patient who has been thus treated early in the disease. The gelsemium is a decided antispasmodic when given in sufficient doses, and in all cases where it is necessary to relax, I know of no better remedy. It has other properties also. It has a great affinity for inflamed mucous surfaces. It is an excellent remedy in gonorrhœa, catarrh of the head, etc. It also has anodyne properties, but not so well marked, except pushed to its physiological action. I think that when combined with morphia, say twenty drops of the gelsemium and one-fourth grain of morphia, it is the best anodyne I ever used. But when combined with opium, or its preparations, it seems to lose its relaxing effect, and the opium its drastic or sickening effect. It is useful in many of the neuralgias. I have known many chronic cases cured, and many others materially benefitted by taking fifteen drops of the tincture in a little water three times daily, about midway between meals. My observation leads me to think it best not to give it just before or after eating.

It is claimed by some that it has parturient properties, but I think the idea originated from the fact that it is one of the best remedies in rigid os uteri. But then it should be followed by a full dose of ergot just as the head begins to press heavily upon the external organs of generation, which is not a bad practice at any time I got into a bad scrape once by neglecting to give the ergot after giving the gelsemium. I came very near losing my patient from hemorrhage.

It is often used now hypodermically. I think that Dr. A. F. Carr, of Goffstown, N. H., was the first to use it in that way. He used the fluid extract in a case of epilepsy, produced from the long continued use of alcoholic stimulants.

I have used it (the tincture) in that way quite frequently for convulsions and neuralgia I use it in ten or twelve drop doses, undiluted, for neuralgia. I inject it over, or as near as possible to, the seat of the pain. If the desired effect is not obtained, I repeat in thirty minutes. A few years ago I was driven to the necessity of using it in that way in a case of puerperal convulsions, though at that time I had never heard or read of its being used in that way. It acted like a charm. I have never given a dose of it that I ever regretted afterwards. My only regret is that I had not known and used it long before I did.

As to its antidotes, I know but little, never having seen any person poisoned by it, but from its action I think strychnia, or nux vomica, or remedies of like action, would prove antidotal. I have had the friends of my patients sometimes to become alarmed because the patient complained of dimness of vision, and appeared so stupid, and to gratify them, I have given the patient a little alcoholic stimulant, or morphia, which very soon proved to their satisfaction.—*Med. Summary*.

---

**Neuralgia.**—Dr. Verneuil reports a case of obstinate neuralgia cured by hyoscyamin, after resection of all the ends of nerves and even amputation had failed to give relief.

